# Development and validation of scale for measuring attitudes towards e-professionalism among medical and dental students: SMePROF-S scale

**DOI:** 10.1186/s12909-021-02879-2

**Published:** 2021-08-23

**Authors:** Marko Marelić, Joško Viskić, Lovela Machala Poplašen, Danko Relić, Dražen Jokić, Tea Vukušić Rukavina

**Affiliations:** 1 School of Medicine, University of Zagreb, Zagreb, Croatia; 2 School of Dental Medicine, University of Zagreb, Zagreb, Croatia

**Keywords:** E-professionalism, Factor analysis, Attitude scale, Validation, Social media, dental students, medical students

## Abstract

**Background:**

Social media permeated everyday life and consequently it brought some changes to behaviour of health professionals. New form of professionalism emerged called e-professionalism depicting professional behaviour while using social media. There are a number of studies conducted in the past several years measuring behaviour of different populations of health professionals on social media and social media sites. Many studies have investigated aspects of e-professionalism of medical or dental students as future health professionals, but there are no validated instruments made for assessing attitude towards e-professionalism of those two populations. Objective of this paper is to validate a newly developed scale for measuring attitudes towards e-professionalism among medical and dental students.

**Methods:**

The original 32-item scale was developed and administered to 411 medical students (RR 69%), and 287 dental students (RR 49.7%). Exploratory factor analysis was used to investigate the existence of underlying factors. Principal component analysis was used as an extraction method with oblimin as selected oblique rotation method. Cronbach’s alpha was used to assess reliability.

**Results:**

Total of 698 student answers entered analysis. The final scale had 24 items that formed seven factors named: ethical aspects, dangers of social media, excluding physicians, freedom of choice, importance of professionalism, physicians in the digital age, negative consequences. Cronbach’s alpha indicating scale reliability was .72. Reliability conducted on each factor ranged from .570 to .877.

**Conclusions:**

The scale measures seven factors of attitude towards e-professionalism and exhibits satisfactory reliability. Based on insights from validation, some possible improvements are suggested.

## Background

The emergence of social networks sites as a new form of digital media and communication channel has brought many challenges for the health system [1–3]. The widespread use of social media affects the context in which professional behaviours are exhibited and how they are interpreted [4].

Professionalism is broadly defined as behaviour in accordance with professional and ethical standards of the profession and can be evaluated through ten components: professional competence, honesty in doctor-patient relationship, health professional-patient privacy, maintaining a proper relationship with the patient, improving the quality of health care, improving the availability of health care, fair distribution of resources, evidence-based knowledge, maintaining patient confidence (prevention of conflict of interest) and professional responsibility [5].

E-professionalism is a derived form of professionalism and can be defined as implementation of traditional principles of professionalism during online activities. Furthermore, it can be defined as a commitment to carry out professional tasks while adhering to ethical principles and care for the patient’s well-being during online social activities [4].

The effort to understand and improve e-professionalism of health professionals on social media sites is visible in the commitment that large number of medical and educational institutions make through various guidelines and advices for online behaviour. Some form of guidelines for e-professionalism are published or available online by a large number of institutions: The American Medical Association [6], The Australian and New Zealand Medical Association [7], The British Medical Association [8] and many others.

First research related to e-professionalism appeared in 2008; in a synthetic review published in 2015 search strategy combine terms “professionalism” with “internet” or “social media” in any field in PubMed, CINAHL and Google Scholar, 941 papers were found, published between 2010 and 2014, of which about thirty that tackle professionalism of healthcare workers on the Internet [9].

The number of users of social media sites grows rapidly so interest in understanding e-professionalism has to follow. Facebook alone currently has over 2.7 billion users active on a monthly basis [10]. If we consider that according to some research about 87% of doctors of medicine have a private account on social networks, and 67% maintain a professional account [11], it is evident that e-professionalism has become an important issue.

In most studies that have health professionals in the sample, the sample is composed of a one specific profession, for example urologists [12], paediatricians [13], surgeons [14], psychotherapists [15] or nurses [16]. A common research question in those studies is how many respondents use social networks and why they use them. Some papers focus on attitude towards social networks, mainly as a review of the benefits and dangers of using social networks for professional or educational purposes.

One study by Chisholm-Burns et al. designed and validated scale for measuring attitudes towards social media professionalism on pharmacy students [17]. After validation, instrument had 22 items that formed five factors named: honesty and integrity, respect for others, accountability, duty, and excellence. Cronbach’s alpha measuring reliability of instrument was .72. Scale covered a broad range of topics: classroom behaviours, conduct such as posting about unprofessional behaviour to social media sites, and the authority of the college/university in monitoring and penalizing students’ social media behaviours.

Even though many studies have investigated aspects of e-professionalism of medical or dental students [18–22], and some investigated attitude towards social media professionalism on similar but different populations [17, 23], so far none of the validated instruments has been made for assessing attitude towards e-professionalism of medical and dental students.

Medical and dental profession have some key similarities that justify comparing them in the field of professionalism. Whilst distinct, dentistry shares many of the ethical foundations of medicine, such as the Hippocratic promise of best interest, confidentiality and respect for autonomy. The dentist/physician–patient relationship, much as is an intimate and personal one, demanding high levels of trust [24].

Regardless of similarities there are some key differences that might result in different attitudes towards e-professionalism. Dentistry is in some aspects considered being closer to cosmetic surgery or business professions, such as low and accountancy. Dentistry is more market oriented, and coupled with different cosmetic treatment options such as bleaching, aesthetic crowns and orthodontics it has created a market based on want rather than needs [24].

The disproportion of medical and dental students’ orientation towards private sector is documenter in UK study on dental undergraduate students who expressed that different expectations are places on dental students regarding SM use compared to medical students given that dentistry is a business [25].

Because of those reasons we believe dental students tend to be more open towards patients on SM, which makes them prone to bigger risk of e-professionalism violation.

The aim of this study is to develop and validate a scale for the assessment of attitude towards e-professionalism in medical students and dental students that could be used not only to measure attitude but also to compare those attitudes of these two populations. Results obtained from this research will be used to create and implement guidelines for e-professional behaviour for medical and dental students during their education, rather than for disciplinary purposes.

## Subjects and methods

The instrument for measuring attitude towards e-professionalism among medical and dental students is part of the larger questionnaire “Exploring the impact of social networks on the professional behaviour of healthcare professionals” conducted for research on project *Dangers and benefits of social networks: e-professionalism of healthcare professionals* (SMePROF project [26]). As a part of the project a quantitative cross-sectional study on the use of SM, attitudes and ethical values of students was carried out in the School of Medicine University of Zagreb, Croatia (UZSM) and the School of Dental Medicine University of Zagreb, Croatia (UZSDM) in the academic year 2018/2019.

Students were informed about the possibility of completing the questionnaire (UZSM 2nd and 5th year, and at the UZSDM students of all 6 years of study) during regular classes. Identification information or IP addresses were not collected to ensure anonymity. All participants were informed in guidelines statement that their participation is voluntary and anonymous. The first question of the questionnaire was informed consent about participation, allowing students that do not want to participate to opt out. Students that gave electronic informed consent on that question entered the sample. Both research and questionnaires were approved by the Ethical board of University of Zagreb School of Medicine, issued on March 22nd 2018 (641–01/18–02/01) and Ethical board of University of Zagreb School of Dental Medicine, issued on February 20th 2018 (05-PA-24-2/2018). All methods were carried out in accordance with relevant guidelines and regulations.

The questionnaire was composed of seven instruments that measured as follows: (1) sociodemographic characteristics and habits of SM usage; (2) knowledge of SM; (3) reasons of SM usage; (4) impression management on SM; (5) security on SM; (6) attitudes towards professionalism; and (7) attitudes towards e-professionalism. This paper covers validation of the seventh instrument that measures students’ attitudes towards e-professionalism.

The instrument was initially composed of 32 items. All items were taken from previous research conducted on similar topics and translated to Croatian language.

Bosslet et al. measured patient-doctor relationship on online social networks with segment dedicated to ethical questions of patient-doctor communication [27]. Therefore, items 1 thru 5 (as shown in Table 1) were taken from their research.

**Table 1:** Average questions scoring, answers range from 1 = completely disagree to 5 = completely agree

	Items	Mean	SD
1	It is ethically acceptable for a physician to communicate with a patient through social media as part of his/her care for patients and the patient healthcare process.^a^	3.30	1.00
2	It is ethically acceptable for a physician to communicate (e.g. share personal messages) with a patient through personal social media account for easier social interaction.^a^	3.25	1.057
3	Social media have the potential to improve communication between a physician and a patient.^a^	3.52	1.00
4	Communication with a patient through social media can be achieved without compromising physician-patient confidentiality.^a^	3.44	1.036
5	It is ethically acceptable for a physician to visit patient social media profile.^a^	3.18	.972
6	It is possible that your potential employer will not hire you or invite you for an interview due to information about you found online.	3.95	.901
7	There is a possibility that your online behaviour might have an impact on perception of others in your profession.	4.09	.800
8	People can make wrong assumptions about you based solely on the content of your post.	4.19	.795
9	You may lose a position you already hold (as an employee or student) due to information about you found online.	3.64	1.006
10	Sharing privileged patient information on social media without their consent is deemed to be inadmissible.	4.68	.747
11	Healthcare professionals should be banned from using social networking software due to too much of a risk.^a^	1.68	1.043
12	Healthcare professionals should be restricted from using social networking software due to too much of a risk.^a^	2.13	1.118
13	I should be able to do whatever I want online.^a^	3.41	1.250
14	The School has no right to interfere in my online activities.^a^	3.86	1.103
15	I believe that my online activities do not affect me as a professional.^a^	3.39	1.228
16	I strongly agree with expectations for professional behaviour and make a conscious effort to comply with them in every aspect of my life.^a^	3.94	.976
17	I know well what constitutes professional behaviour and what is expected of me as a current/future professional.^a^	4.09	.850
18	High-level professional behaviour should also be expected of students from the very beginning of their studies.^a^	3.52	1.196
19	Guiding patients to online information is a new responsibility of physicians in the digital age.^a^	2.81	1.283
20	As a medicine / dental medicine graduate, it is my obligation to keep abreast with the current trends in the use of social media.^a^	3.79	1.100
21	One of the responsibilities of a teacher is to counsel students on the appropriate use of social media.^a^	2.98	1.315
22	Professionals cannot actually fully relax.^a^	3.42	1.182
23	Social media have removed protection of professionals against the public.^a^	3.34	1.143
24	It is not always possible to maintain professionalism in online activities.^a^	3.68	1.027

Items marked with asterisk (^a^) were measured without neutral answer

In similar research, White et al. measured attitudes to guidelines relating Facebook use [28]. Total of 21 items were taken from their research, out of which 18 entered the final scale (6 thru 18 and 22 thru 24), and 5 items that didn’t (25, 26, 29, 30, 31 as shown in Table 2).

**Table 2:** Items excluded after validation

	Items	Reason for exclusion
25	People have the opportunity to post photos and document aspects of their professional life which would otherwise remain private.	Low saturation on all factors
26	A little leniency should be shown if unprofessional behaviour occurs in the first years of professional education.	Not contributing to one factor; low inter-item correlation;
27	The risks of social networking software greatly overweigh the benefits.	Equally low contribution to all factors
28	Patients use social media to get medical / dental information.	Ambiguous direction of item, not contributing to one factor
29	Professionalism in online activities is as important as in traditional (offline) environments.	Low contribution to multiple factors
30	I believe discussion on online professionalism to be more important for my profession than for any other.	These two items form specific dimension that stays uncorrelated with rest of instrument, suggesting they do not measure a part of e-professionalism
31	I believe discussion on online professionalism to be more important for my healthcare profession (physicians of different specialties) in respect to any other profession.	
32	The benefits of social media overweight the risks of their use.	Equally low contribution to multiple factors

Three items (19 thru 21) measuring the aspect of responsibility towards guiding patients on social networks were taken from the study of Kitsis et al., as well as two items that were not included in the final version (27, 28 as shown in Table 2) [2].

In order to assess the developed scale, it was administered to 714 students at the School Medicine and School of Dental Medicine, University of Zagreb, Croatia in the period from 10th of November 2018 to the 4th of January 2019. After screening results, 698 answers entered the analysis.

Before administering, content validity was reviewed and affirmed by the research team of experts from various scientific fields: sociologist, doctor of medicine, doctors of dental medicine, psychiatrist and communicologist. Following the example of Ichikawa et al., six researchers repeatedly checked whether items represented e-professionalism, whether item content was suitable for judging attitude towards e-professionalism of dental and medical students, and whether concept of e-professionalism was covered in the entire scale [29]. The researchers agreed that all items included in this scale reflected attitude towards e-professionalism.

Data analysis was performed using IBM SPSS statistics 25. Demographic data were summarized as descriptive statistics.

Construct validity was investigated using exploratory factor analysis, followed by scale reliability analysis using Cronbach’s alpha coefficient of the internal consistency. In an iterative process of repeating dimensionality analysis and reliability analysis after each item that was discarded instruments was improved and validated. Internal consistency was investigated for each factor and for the instrument as a whole.

As the extraction method, principal component analysis was used with *oblimin* as selected oblique rotation method.

## Results

### Study sample

Of the 714 collected students’ responses a total of 698 entered analysis, 411 from second and fifth-year medical students (RR 69%), and 287 from first to sixth year dental students (RR 49.7%); mean age of 22, range 18 to 30 years, 74% of the students were female and 26% were male.

In order to provide a better picture about this convenience sample three variables were measured: active or passive type of usage, frequency of access and type of device most used.

### Development, reliability and validity of SMePROF-student version (SMePROF-S) scale

Construct validity was tested with exploratory factor analysis that was conducted on 32-item scale. Kaiser-Meyer-Olkin measure (.771) and Bartlett’s test of sampling adequacy (χ^2^ = 6873.78, df = 496, *p* = 0.000) shows that dimension reduction is applicable on this data. Initial factor analysis extracted nine factors that cumulatively explained 62.8% of total variance. Reliability of the initial 32-item scale was α = .765.

One item (25 – *People have the opportunity to post photos and document aspects of their professional life which would otherwise remain private)* was removed because it did not measure attitude and it saturated equally on multiple factors. In following iteration, item (26 – *A little leniency should be shown if unprofessional behaviour occurs in the first years of professional education*) was removed because it didn’t have adequate saturation on neither of factors, probably because overall very positive score on this sample (about 85% of students agreed). Additional six items were deleted because they failed to load on any of the factors.

Final scale consists of 24 items and factor analysis produced seven factors explaining 65.53% of cumulative variance that can be logically described (Table 3). Cattel’s scree test confirmed the same number of factors as the Kaiser criterion (eigenvalue greater than one).

**Table 3:** Principal components analysis with corresponding Cronbach α coefficients for each factor

		Factors						
		Ethical aspects	Dangers of SM	Excluding physicians	Freedom of choice	Importance of professionalism	Physicians in the digital age	Negative consequences
Factors	Items	1	2	3	4	5	6	7
Ethical aspects	It is ethically acceptable for a physician to communicate with a patient through social media as part of his/her care for patients and the patient healthcare process.	**.885**	.020	−.067	.030	.008	.162	.022
	It is ethically acceptable for a physician to communicate (e.g. share personal messages) with a patient through personal social media account for easier social interaction.	**.870**	.072	−.080	.076	.006	.136	.018
	Social media have the potential to improve communication between a physician and a patient.	**.865**	.100	−.127	.024	.059	.243	.021
	Communication with a patient through social media can be achieved without compromising physician-patient confidentiality.	**.832**	.041	−.101	.013	.012	.191	.078
	It is ethically acceptable for a physician to visit patient social media profile.	**.625**	.086	−.030	.122	−.021	.108	.067
Dangers of SM	It is possible that your potential employer will not hire you or invite you for an interview due to information about you found online.	.109	**.838**	−.073	−.040	.065	.101	−.114
	There is a possibility that your online behaviour might have an impact on perception of others in your profession.	.037	**.827**	−.049	−.025	.185	.120	−.177
	People can make wrong assumptions about you based solely on the content of your post.	.050	**.821**	−.102	−.016	.176	.105	−.103
	You may lose a position you already hold (as an employee or student) due to information about you found online.	.040	**.773**	.018	−.051	.087	.124	−.067
	Sharing privileged patient information on social media without their consent is deemed to be inadmissible.	.108	**.588**	−.379	.102	.166	.044	−.028
Excluding physicians	Healthcare professionals should be banned from using social networking software due to too much of a risk.	−.091	−.140	**.896**	.034	.076	.016	−.131
	Healthcare professionals should be restricted from using social networking software due to too much of a risk.	−.116	−.028	**.870**	−.101	.122	.108	−.219
Freedom of choice	I should be able to do whatever I want online.	.074	−.010	−.088	**.788**	−.063	−.040	−.198
	The School has no right to interfere in my online activities.	.021	.023	−.245	**.733**	−.069	−.144	−.324
	I believe that my online activities do not affect me as a professional.	.057	−.057	.113	**.687**	.168	.059	.076
Importance of professionalism	I strongly agree with expectations for professional behaviour and make a conscious effort to comply with them in every aspect of my life.	.012	.138	.067	.044	**.874**	.045	.012
	I know well what constitutes professional behaviour and what is expected of me as a current/future professional.	.019	.151	−.025	.139	**.824**	−.059	−.076
	High-level professional behaviour should also be expected of students from the very beginning of their studies.	.000	.126	.190	−.130	**.736**	.221	.035
Physicians in the digital age	Guiding patients to online information is a new responsibility of physicians in the digital age.	.266	.058	.073	.009	−.013	**.768**	−.060
	As a medicine / dental medicine graduate, it is my obligation to keep abreast with the current trends in the use of social media.	.165	.076	−.130	.073	.088	**.759**	−.153
	One of the responsibilities of a teacher is to counsel students on the appropriate use of social media.	.068	.191	.181	−.140	.090	**.649**	.028
Negative consequences	Professionals cannot actually fully relax.	−.105	.160	.145	.034	−.005	.093	**−.831**
	Social media have removed protection of professionals against the public.	−.063	.097	.209	.015	.088	.082	**−.798**
	It is not always possible to maintain professionalism in online activities.	.042	.072	.001	.339	−.018	.044	**−.653**
Cronbach α coefficients		.877	.829	.823	.601	.748	.570	.680

Reliability analysis was conducted on final scale and Cronbach coefficient was α = .72, furthermore, reliability of each factor was conducted separately and α coefficients ranged from .570 to .877 as it is shown in Table 3.

The first factor is called *Ethical aspects* and five items in this dimension measure attitudes towards ethical aspects of communicating with patients via social networks. It encompasses whether it is ethically acceptable to communicate with patients through social networks and does social media have the potential to improve communication between physician and a patient.

The second factor is called *Dangers of SM* and it is composed by five items measuring perceived negative impact of social networks on future (or current) employment, as well as possibility that people can make wrong assumptions based on the content of one’s posts.

The third factor named *Excluding physicians* measures attitude towards prohibiting or restricting usage of social networks to medical workers.

The fourth factor named *Freedom of choice* is formed by three variables measuring independence of acting (and posting on social media) from college rules and social rules, suggesting that online activities do not affect their professional performance.

The fifth factor named *Importance of professionalism* is connecting e-professionalism to “offline” professionalism (or professional behaviour as already known concept). Factor is formed by items describing need for professional behaviour as well as self-assessment about what constitutes professional behaviour.

The sixth factor, *Physicians in the digital age*, measures attitude towards positive inclusion of social networks in the medical profession, making physicians and medical teachers responsible for education and guidance of patients and students in tenants of “correct” type of network usage.

The seventh factor called *Negative consequences* is formed by three items and encompasses overall negative attitudes toward changes that social networks brought; namely in protection of physicians from public and inabilities to fully relax and maintain professional image.

Second-order factor analysis conducted on factor scores from obtained seven factors was used to further describe the dimensionality of the instrument. Seven factors are grouped in four second-order factors, as it is shown in Table 4. It’s important to notice that factor *Excluding physicians* does not group with other factors.

**Table 4:** Second order factors analysis, a structure matrix

	Second order factors			
Factors	1	2	3	4
Ethical aspects	**.803**	−.284	−.034	.056
Physicians in the digital age	**.732**	.333	.017	−.240
Excluding physicians	−.038	**.869**	.028	.005
Negative consequences	.040	−.258	**.772**	.180
Freedom of choice	.062	−.294	**−.741**	.136
Dangers of SM	.161	−.183	−.105	**−.771**
Importance of professionalism	.000	.178	.044	**−.707**

Inspection of average question scoring provided in Table 1, can be used to further clarify the attitudes of students in this sample. Since this scale was part of the larger questionnaire with multiple instruments, some items were measured on a five-point scale, and some were measured on a four item scale (without the neutral answer). That is the reason for the somewhat larger standard deviation on later type of items. The highest degree of agreement was on the item 10 – *Sharing privileged patient information on social media without their consent is deemed to be inadmissible.* Suggesting that there is no ambiguity about ethical perspective of dealing with privileged information, also supported with a high degree of agreement with item 17 that states that they know what *constitutes professional behaviour and what is expected of me as a current/future professional*. However, attitude toward teachers being one responsible for counselling students on the appropriate use of social media did not have such uniform consent and using sanctions for misbehaviour (like banning or restricting medical professionals from social networks) were items with the largest degree of disagreement.

## Discussion

As far as authors know this is the first scale designed to investigate attitudes towards e-professionalism on student population of medicine and dental medicine.

Scale demonstrated acceptable reliability score (α = .72). Values of Cronbach alpha used in this study in order to demonstrate degree of internal consistency can lie between 0 and 1, and values between 0.7 and 0.9 are interpreted as acceptable reliability [30].

Final scale that was developed through validation is composed of 24 variables and yielded seven factors that collectively explained 65.53% of cumulative variance. Reliability of each factor was conducted separately and α coefficients ranged from .570 to .877. Coefficients that have lower values than 0.7 are likely results of a small number of items that form them (factor P*hysicians in digital age* is formed by only two variables and has lowest consistency with α = .570). Authors recommend increasing the number of items measuring that specific dimension in order to improve scale.

Several factors found in this scale match some of the tenants of (traditional) professionalism defined by ABIM foundation [5]. This can be understood as a form of verification of definition of e-professionalism, since we had no empirical guarantee what exact factors would reflect in phenomenon of e-professionalism. Protecting a patient’s privacy and maintaining proper relationships with patients defined as tenants of professionalism can be seen in e-professionalism scale as factors *Dangers of SM* and *Ethical aspects*. Furthermore, improving the quality of healthcare can be seen in factor *Physicians in the digital age*. As it could be expected, other factors are specific for e-professionalism.

It’s interesting that item 10 – *Sharing privileged patient information on social media without their consent is deemed to be inadmissible* saturated on factor *Dangers of SM* rather than *Ethical aspects*. It is possible that sharing patient information is regarded as a direct danger of social networks, that can lead to further consequences described by other items composing first factor (for example, losing a job), rather than question of ethics. In other words, it’s pragmatic question that can directly impact student’s life and not moral or ethical opinion that indirectly guides decisions. It is also possible that students responded this way because it’s preferred answer and acceptable behaviour, but that doesn’t mean they will behave accordingly. This is known as “cognitive dissonance” and it’s a disconnect between what student thought they *would* do versus what they thought they *should* [13, 31]. This inconsistency between attitudes and actions has been observed also elsewhere [32, 33]. However, measuring just e-professional behaviour in students can result in incomplete and problematic results since students don’t have regular and uniform contact with patients. They have some contact during practical classes, but they lack practical experiences and quantity of doctor-patient relations. Measuring attitude therefore overcomes this problem, and even though it’s not perfect prediction of behaviour, it’s a sufficient predictive indicator that can be used for creation of educational policy and guidelines.

Factor *Excluding physicians* requires further testing. It is possible that the formulation of items was not tailored for students but rather physicians with existing experience of dealing with patients on social networks.

Considering average question scoring, results suggest negative aspects of social media (or dangers of social media) are more emphasized than benefits in this sample. It is visible that students in the sample are very aware of the impact of social media on future employability.

This finding is consistent with White et al. research [28]. It’s possible that negative consequences and social media related scandals are far more visible and emphasized in the media than positive examples and potential benefits of social networks. White et al. hypothesized that students face dilemma between sharing personal content that could improve their popularity and reducing risk of unwanted attention, for example from employer. Based on results of this study, in terms of “push” or “pull” reasons, we could say that for students in our sample “push” reasons outweighed “pull”.

Bosslet et al. found that the majority of respondents found ethically unacceptable to visit the profiles of patients on SM. Additionally, a majority of students in the sample did not agree it was ethically acceptable to interact with patients on SM, either for social or patient-care reasons [27]. However, in our research respondents were much more ambiguous, which can be seen from average scoring of items 1 through 5 (Table 1) that are much closer to neutral values than agreement.

Some tenants of e-professionalism of medical and dental students are consistent with tenants found in scale for measuring pharmacy student’s attitudes towards social media professionalism [17]. For example, topic that tackles role of university in dealing with e-professionalism measured in Chisholm-Burns et al. scale is consistent with factor *Freedom of choice* in this instrument. Also, the role of social media in future employability is occurring topic that is unavoidable when assessing attitudes of students.

Students in sample on average stated that they understand what constitutes professional behaviour and clearly expressed rebuff about using sanctions for online behaviour. Even though they stated that they know what constitutes professional behaviour there is some ambiguity underlined because there was not unanimous response on whether it’s acceptable to visit patients’ profiles or not. This suggests that students in the sample had rather individual concepts of professionalism and what is permissible or not. This clearly calls for the need for teaching e-professionalism during formal education and providing them with an unambiguous perspective on e-professionalism.`

### Limitations

This study has several limitations. First, all items in this scale were taken from previous researches and therefore some theoretical concepts that would have emerged from traditional operationalization of theoretical concepts might be omitted. Although larger number of factors can be expected in first versions of scale development, it is possible some dissipation of item saturations is the result of that.

Second, *Excluding physicians* being a dimension itself both in factor analysis and second order factor analysis suggests that there is a possibility that as a dimension measured with these particular items is not a domain of e-professionalism, but rather a different topic that requires its own measuring instrument. Regardless, it’s an important theoretical part of e-professionalism because it covers students’ autonomy and freedom to use SM regardless the profession. Students can position their attitude in relation to extreme measure of banning or restricting usage of SM to healthcare workers in order to reduce space for unprofessional behaviour. Therefore, removing it completely would damage the content validity of the scale so it was not removed. Perhaps in some future iteration of this scale these two items could be rewritten in order to reflect on particular type of risks instead of the general statement and therefore improving measurement.

Third, this scale was part of a larger questionnaire and items were measured within different parts, resulting in difference in range of rating scale (four-point and five-point). Results of factor analysis were not impaired, but caution is recommended in interpreting average item scoring. Four-point items were recoded into five-point scale without neutral value (value 3 into 4; value 4 into 5). That is reason of increase in standard deviation in those changed items (visible in Table 1). For future usage of this scale we recommend unifying all items on five-point scale.

Fourth, the questionnaire was conducted on Croatian language and although questionnaire was translated by English language experts some of the meaning can be lost or changed. For delicate process of finding underlying dimensions that epistemological differences could impact comparability with other scales.

Fifth, all questions were mandatory in order to avoid missing values. That could result in larger degree of respondents withdrawing from research without completing questionnaire. Unfinished questionnaires could not be collected in online survey platform used (Google Forms).

## Conclusion

SMePROF-student version (SMePROF-S) scale for measuring attitude towards e-professionalism among medical and dental students proved to be valid and reliable. Original version of scale has undergone substantial changes during validation process and final version is composed of 24 items that measure seven dimensions of e-professionalism.

This scale can be used to assess and compare medical students and dental medicine students regarding their attitude towards e-professionalism. This is the first validated scale designed to measure attitude towards e-professionalism on this specific population and therefore there is room for improvements in the future iterations.

## Data Availability

The datasets used and/or analysed during the current study are available from the corresponding author on reasonable request.

## Abbreviations

RR: Response rate
SMePROF: Social Media e-Professionalism
SM: Social media
UZSDM: University of Zagreb School of Dental Medicine, Croatia
UZSM: University of Zagreb School of Medicine, Croatia
